# Understanding Barriers to Human‐Wildlife Coexistence: Evidence From Southern Sri Lanka

**DOI:** 10.1002/ece3.73859

**Published:** 2026-06-30

**Authors:** Anuradhi Dulangi Jayasinghe, Georgette Leah Burns, Duan Biggs

**Affiliations:** ^1^ School of Environment and Science, Griffith Institute for Human and Ecosystem Resilience Griffith University Brisbane Queensland Australia; ^2^ Centre for Adaptable Western Landscapes & School of Earth and Sustainability Northern Arizona University Flagstaff Arizona USA; ^3^ Centre for Sustainability Transitions Stellenbosch University Stellenbosch South Africa; ^4^ Rob Walton School of Conservation Futures Tempe Arizona USA

**Keywords:** coexistence barriers, coexistence strategies, human‐wildlife coexistence, human‐wildlife conflict, human‐wildlife relationships

## Abstract

Human‐wildlife coexistence (HW‐coexistence) is a central goal of conservation. However, efforts to achieve coexistence have largely focused on mitigating human‐wildlife conflict, while overlooking the underlying barriers to promote coexistence. We aimed to understand barriers to HW‐coexistence by focusing on farming communities interacting with elephants ( *Elephas maximus maximus* ) in Sri Lanka. A mixed‐method ethnographic study was conducted using participant observation, focus group discussions and transect walks in Walsapugala village in Southern Sri Lanka. In this study, socio‐political drivers, institutional and structural challenges and power and corruption were identified as themes of barriers to coexistence. Our results revealed that conflicts extend beyond farmer‐elephant interactions to encompass other wildlife species and wildlife governing authorities. Despite their importance in influencing barriers to coexistence, these conflicts have received limited attention in human‐wildlife interaction (HWI) literature. We argue that future research must move beyond an exclusive focus on isolated technical solutions for conflict mitigation. Instead, it should prioritise understanding the hidden barriers to coexistence, including the improper implementation of mitigation strategies driven by socio‐political drivers, institutional and structural challenges and issues of power and corruption.

## Graphical abstract

We investigated the barriers to coexistence with wild elephants ( *Elephas maximus maximus* ) as experienced by farmer communities in Southern Sri Lanka. Through an ethnographic study, we identified socio‐political drivers, institutional and structural challenges, and power and corruption as the main themes of barriers to coexistence. We argue that a meaningful pathway toward coexistence requires a deeper understanding of the hidden barriers to coexistence ‐ such as corruption and institutional and structural challenges as identified in our study ‐ that hinder effective implementation of conflict mitigation strategies.

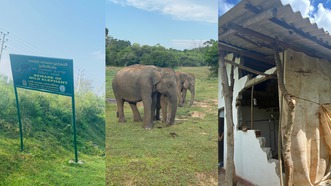

## 1 Introduction

Human‐wildlife coexistence is ‘a dynamic but sustainable state in which humans and wildlife co‐adapt to living in shared landscapes’ (Mekonen 2020, 9). To achieve this state, Mekonen (2020) maintains that human interactions with wildlife are governed by institutions that effectively ensure three things: (1) long‐term wildlife population persistence, (2) social legitimacy and (3) tolerable levels of risks. Although human‐wildlife interactions are often posited as a dichotomy of conflict versus co‐existence, coexistence does not mean the absence of conflicts (Madden and McQuinn 2015; Pooley et al. 2020; Gao et al. 2023). Conservation tools such as protected areas promote a dualism between humans and wildlife by allocating specific areas where humans and wildlife are separated. This separation can instil a notion that humans and animals are in opposition, exacerbating the tension between conflict and coexistence and assuming that coexistence is only achieved in the absence of conflict (Jolly and Stronza 2024). Frank and Glikman (2019) argue that societal views on wildlife define the outcomes of human‐wildlife interactions. Thus, depending on the context, interactions can be perceived as coexistence (e.g., respect for wildlife and deep affiliation with nature), neutrality (i.e., mixed attitudes/behaviours and remains indifferent towards wildlife issues) or conflict (e.g., retaliatory killing of wildlife). The field of human‐wildlife conflict and coexistence (HWCC) grew from an anthropocentric concern about real or perceived threats to people's interests then, from the mid‐1990s, navigated towards protecting valued species from people (Treves and Santiago‐Avila 2020).

Globally, tensions at the human‐wildlife interface are driven less by wildlife behaviour alone and more by intensified anthropogenic activities (e.g., human settlement) in and around protected area boundaries. Conflicts with large mammals, such as elephants, occur predominantly outside protected areas, within cultivated and residential landscapes where human presence is dominant (Bhandari et al. 2020). In remote forest‐fringe settings, such as Talkharka in India's East Sikkim district on the fringes of the Pangolakha Wildlife Sanctuary, conflicts between cardamom farmers and wildlife (e.g., Himalayan Palm Civets, Wild Boar, for example) have found to be increasing (Ghosh et al. 2023). In southern African contexts, proximity to protected areas has intensified negative interactions with wildlife—such as ‘crop destruction and loss of livestock to wild predators or disease and often human mortality’ (Wahab et al. 2021, 21). However, although numerous studies have documented the conflicts affecting communities adjacent to protected areas, an in‐depth investigation into the social, economic and governance‐related barriers for coexistence remains largely overlooked.

Tolerance and risk are important concepts in the definition of HW‐coexistence because ‘coexistence does not imply there is no risk; rather, it requires tolerance of risks and the management of risks such that they remain within tolerable limits’ (Pooley et al. 2020, 785). Hence, issues that affect human tolerance of wildlife‐related risks are critical to include in our investigation of the barriers to coexistence, and the promotion of coexistence must include strategies grounded in multiple, interrelated issues.

Focus on implementing conflict mitigation strategies alone may not be sufficient to foster coexistence, particularly when underlying and often hidden barriers remain unaddressed. For instance, financial compensation schemes for wildlife‐related losses (Badola et al. 2021; Yeshey et al. 2024) are frequently used to increase community tolerance towards both damages and the wildlife responsible (Kansky et al. 2020; Hemminger et al. 2025). However, such approaches can exacerbate conflict when issues such as unequal power relations, corruption or bribery in compensation administration, and lack of trust undermine their effectiveness (Bulte and Rondeau 2007). The success of compensation mechanisms depends not only on community values and perceptions but also on how transparently and equitably these strategies are implemented (Hemminger et al. 2025). Therefore, coexistence requires addressing less visible structural barriers and underscores the importance of researchers and managers actively involving local communities in the design and implementation of conservation strategies to ensure that all obstacles to coexistence are adequately identified and addressed. To understand the barriers to HW‐coexistence, we focused on human‐elephant interactions in Walsapugala village in Southern Sri Lanka as a case study.

### 1.1 Study Context: An Introduction to the Mahaweli Program and Walsapugala Village

Asian elephants ( *Elephas maximus* ) were declared endangered in 2008, mainly due to the loss of habitats globally (IUCN 2008). Sri Lanka, a global biodiversity hotspot, is home to approximately 10% of the remaining Asian elephant population while encompassing only 2% of the Asian elephant range (Rathnayake et al. 2022). Sri Lankan elephants ( *Elephas maximus maximus* ) are an endangered subspecies of the Asian elephant (Gunawansa et al. 2023), and continued habitat decline has driven them into increasing contact with humans, especially in cultivated lands. Consequently, the balance of human‐elephant coexistence in Sri Lanka is under threat. Deaths of both elephants and humans due to conflict are rising (Begum 2024), with 433 elephant and 145 human fatalities recorded in 2022 (Shazuli 2023). Sri Lanka has the highest recorded number of annual elephant deaths and the second‐highest human deaths due to human‐elephant conflicts in the world (Gunawansa et al. 2023).

In Sri Lanka, wild elephants come under the jurisdiction of numerous institutions, including the Department of Forest Conservation, the Department of Wildlife Conservation (DWC), the Irrigation Department, the Land Reform Commission and the Mahaweli Authority (Centre for Environmental Justice (CEJ) 2024). No coordinated elephant management programs exist between these organisations.

Walsapugala village, in Hambantota district, is part of the largest irrigation‐based agricultural program in Sri Lanka: the Mahaweli Development Program (MDP) (Paranage 2019). Designed in 1968 by the former government with the technical support of UNDP/FAO, the MDP focused on rural agricultural and settlement development of the dry zone in Sri Lanka by harnessing natural water resources from the central hills of the country (Mahaweli Authority of Sri Lanka (MASL) 2021). Walsapugala village is located in the Mayurapura Mahaweli block of Hambantota district.

**FIGURE 1:**
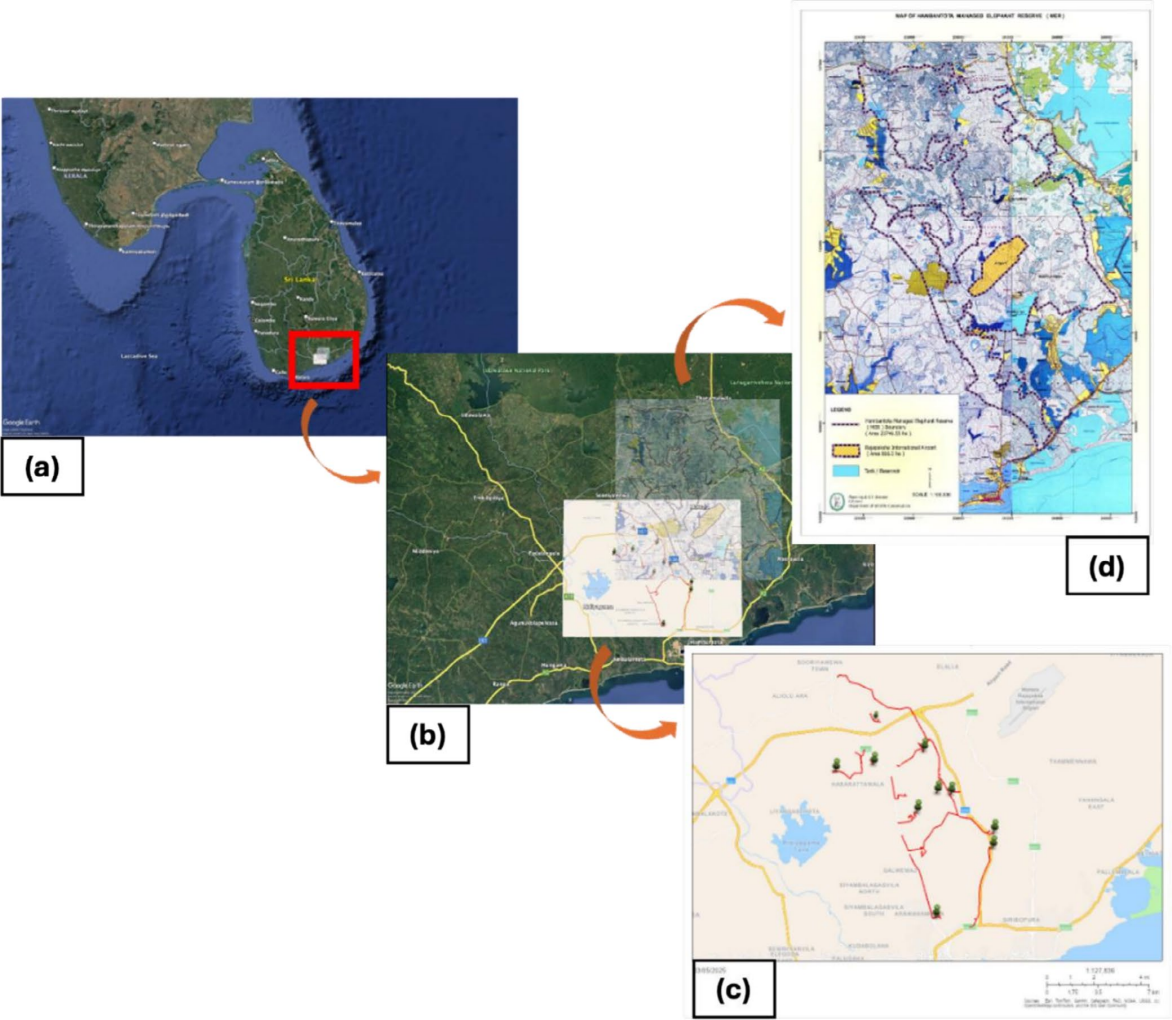
Four maps of the study location. (a) A map of Sri Lanka highlighting Walsapugala village. (b) ArcGIS map of the study area and the proposed MER overlayed in Google Earth Pro. (c) A map showing pinned locations where FGDs and transect walks took place. (d) A map of the proposed MER.

From 2005 to 2015, the Hambantota district experienced massive transformation through the Greater Hambantota Development Program. The population of 10,000 people, after independence in 1948 (Mariyathas et al. 2016), was estimated to have grown to 680,000 by mid‐2023 (Register General's Department Sri Lanka 2023). The district now has a harbour, an international airport, a high calibre cricket stadium and highways. Before the project implementation that heralded the changes, Sri Lankans were promised that this rural area would become the epicentre of the country's economic, social and environmental development (Mariyathas et al. 2016). However, the airport rarely commutes flights, the cricket stadium is seldom used, and the harbour barely functions. These massive development initiatives in Hambantota displaced farmers and disturbed habitat for many wildlife species, with elephants the most severely affected. The situation worsened when the displaced humans were involuntarily relocated into elephant habitats, increasing human‐elephant interactions and conflict. A 23,000 ha managed elephant reserve (MER) was suggested in 2009 (Wickramasinghe 2024) in response to human elephant conflict (HEC) related issues raised by the Department of Wildlife (see Figure 1d). However, MER was not gazetted until 2021 (Rodrigo 2021), following a 108‐day‐long protest in Walsapugala village, and has not yet been enforced. Illegal activities (e.g., rock quarries) happen within this proposed reserved area (Perera 2021) and habitat loss forces wild elephants to search for food and water sources from the nearby villages, like Walsapugala, (see Figure 1b).

## 2 Methodology

### 2.1 Focus Group Discussions, Participant Observations and Transect Walks

An ethnographic study was conducted in Walsapugala village in Hambantota District in Southern Sri Lanka from November 2024 through February 2025. Ethical clearance for the research was obtained from Griffith University's Human Research Ethics Committee (Ref No: 2024/297). Using an inductive approach (Oberlack et al. 2019; Pacheco‐Romero et al. 2021), the questions during the focus group discussions, transect walks and observations were not tied to any preconceived notions (Appendix A). Participant observations and focus group discussions (FGD) were conducted across eight *Mahaweli* Units of the *Mayurapura Mahaweli* Block. Table 1 provides information about the FGD participants as represented by different units of the Mayurapura Mahaweli Block of Walsapugala village. Discussions in the eight, open‐ended FGD (*n* = 14 average number of participants per FGD; total *n* = 113) were based on the research question of understanding the barriers to coexistence with elephants.

**TABLE 1:** Number of participants in FGD representing farmer families from selected units in Mayurapura Mahaweli block across Walsapugala village.

System	District	Divisional secretariat	Mahaweli block	Mahaweli Units of the Walsapugala village	Number of farmer families	Number of FGD participants	% of FGD participants
Walawa System	Hambantota	Sooriyawewa	Mayurapura	Namadagaswewa	917	0	0
				Andarawewa	435	19	4.4
				Thelawilla	200	0	0
				Ruhunupura	60	10	16.7
				Karuwalawewa	65	18	27.7
				Bolhinda	280	7	2.5
		Hambantota		Galwewa	275	14	5.1
				Bellagaswewa	250	15	6
				Ranamayurapura	20	0	0
				Katuwewa	80	15	18.8
				Thissapura	30	15	50
Total					2612	113	4.3

The FGD participants were purposely selected with the help of key informants, who included leaders of the farmer associations of the village. Participants included any willing household member who was over 18 years old, had lived in the area for at least 10 years, engaged in farming activities and had experience interacting with wild elephants. Each discussion included a mixed representation of men and women and lasted for 2–3 h. The FGDs were conducted at houses selected in the village.

Each FGD was followed by a transect walk through farmland and residential areas damaged by elephants, where data was collected during informal discussions and observations. Volunteer guides for these transect walks were community members (3–4) who also participated in the FGDs. Here data was collected from informal discussions and observations.

Transect walks in Walsapugala village were tracked through GAIA GPS trail maps and transmitted to ArcGIS to develop the map in Figure 1c, which also shows locations where FGDs took place. In some areas, these transect walks were supported by tractors and trishaws when the pathways were too muddy/slippery to walk. Transect walk times varied from 1 to 2 h depending on the area covered.

The FGDs and discussions during the transect walks were conducted in the Sinhalese language and audio recorded with participant consent. The observations were recorded through photos and videos or written down to support the analysis. Figure 1 shows four maps of the study area.

### 2.2 Coding

**FIGURE 2:**
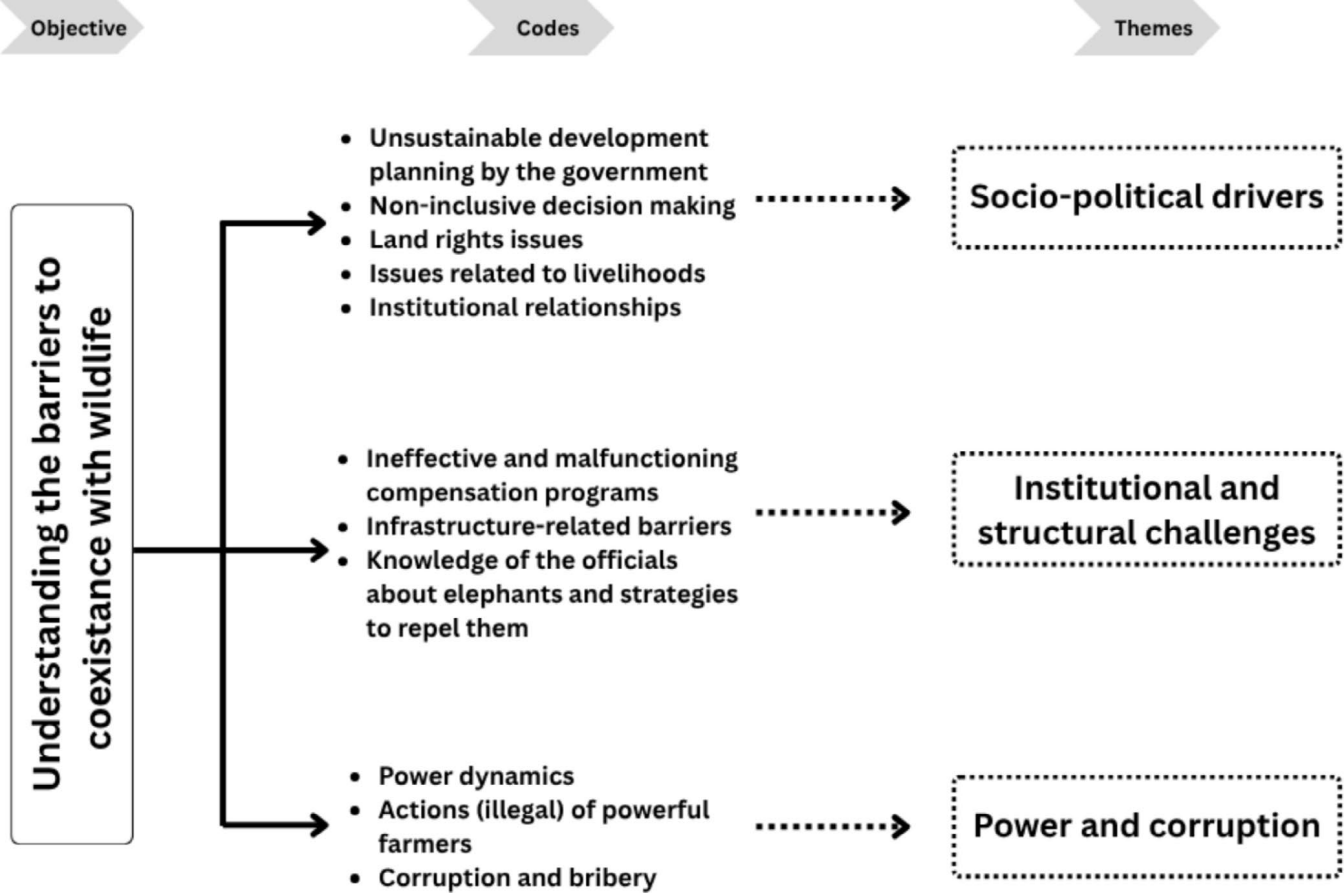
Themes developed using the identified codes of barriers to coexistence.

The audio recordings of the discussions and observations were transcribed and translated into English. The transcribed and translated data were coded through NVIVO 14 (release 14.24.3), to develop initial codes to understand the barriers to coexistence, inductively as arose during the analysis based on their meanings and patterns (Appendices A, B, C). Initial open coding was conducted line‐by‐line to identify concepts related to barriers to coexistence as they emerged from data, without applying predefined categories. Codes were generated based on recurring meanings, issues and patterns across the dataset and included, for example, *institutional relationships, land use rights, resilience, elephants as intelligent creatures* and *place attachment*. Following open coding, related codes were iteratively compared and grouped through manual axial coding to identify broader conceptual linkages. This allowed organisation of the codes into higher‐order categories that reflected shared underlying barriers to coexistence. These categories were refined through repeated engagement with the data and resulted in three overarching themes of barriers to coexistence as presented in the results section below (Figure 2).

The development of themes was informed by ethnographic interpretation by the researcher (Byrne 2021). Data analysis followed an interpretive qualitative approach (Schwartz‐Shea and Yanow 2012) aimed at understanding participant responses in relation to their lived experiences of interacting with elephants, including how they perceive risks and institutional support, for example. This was combined with reflexive thematic analysis, a widely used method in the social sciences that involves iterative engagement with data to identify patterns of meaning across the dataset (Braun and Clarke 2019). This also included maintaining analytic memos during coding, revisiting interpretations through repeated readings of data and ensuring that themes were grounded in participants' responses.

### 2.3 Limitations of the Study

There were two key limitations in this study. First, because data was only collected from local farmers, the entire local population is not represented, and the barriers to coexistence are explained only through the farmers' perspectives. However, these participants also provided information about barriers faced by wildlife officers. Second, the analytical data is entirely qualitative and relies on subjective interpretation by the authors. However, the methodology (FGDs, transect walks and observations), discussion questions used, and the codes constructed initially in the analysis can be used in future research related to HW‐coexistence. Despite the subjective nature in the analysis, underlying aspects of the barriers to coexistence highlighted in the discussion are likely to be found in the context of the Global South especially where conflicts between humans and wildlife exist.

## 3 Results

The results revealed three barrier themes: (1) socio‐political drivers, (2) institutional and structural challenges and (3) power and corruption. These themes are discussed below, with examples drawn from different FGDs (these examples are direct quotes from discussions, followed by the FGD number; see Appendix C). Figure 2 shows how these themes were developed by collating different codes identified during the analysis phase.

### 3.1 Socio‐Political Drivers

The barriers identified under the theme of socio‐political drivers are multifaceted. Crop cultivation is the main livelihood of these farmers. However, according to the discussants, low cultivation variety, coupled with the lack of established markets to sell their harvests, and continued crop raiding by elephants (as well as other wildlife such as toque macaques), are key barriers for them to coexist with elephants. The farmers also emphasised that the infrastructure developments that took place in the area were non‐inclusive and short‐sighted—and the root cause of existing HEC.

Walsapugala farmer communities rely on a small variety of vegetable cultivation such as brinjal, chillies and tomatoes grown in backyards mainly for subsistence and bananas and paddy to sell in the market. Farming in their own lands is their main source of income, but working as labourers in nearby farmlands is also increasingly popular, mainly due to the absence of markets to sell their products. Discussions with Walsapugala farmers revealed that their issues related to living with elephants were exaggerated by other issues that related to their livelihoods. For example, when families abandon their farmlands due to elephant conflicts, those lands eventually become feeding grounds for elephants. As a consequence, the effort for remaining farmers to protect their cultivations from elephants increases. Moreover, elephants are not the only wildlife of concern; toque macaques, peacocks, porcupines and wild squirrels also damage crops:

We can't even grow a chilli pod for household use, which has even been raided by monkeys. We can't go out for a full day, by the time we come back, our crops will be destroyed by monkeys in five minutes. (FGD 1).

When asked about the increase in such encounters, some complained that these animals were dumped into their village from nearby tourist hotspots to avoid tourist complaints about wildlife attacks:

The area where those animals were dumped from, Kataragama, still has enough food for them. When their food sources are fading away, will they come to these areas? (FGD 2).

These farming communities witnessed one of the largest infrastructure developments in Sri Lanka's history under the Greater Hambantota Development program. Although these families lived in their lands for generations, they lack formal documentation or land titles to prove ownership, particularly among those displaced by development projects. Farmers complain that they been excluded from the development planning and the habitats of both them and wildlife were sold to foreign investors. During the discussions, farmers showed anger towards government officials for being insensitive about land occupancy by farmers and wildlife.

For those foreign investors, these officials have shown the best fit area, which is highly impacted by elephants and used as a corridor. The officials, by any means, want to bring the investment during their period to reap the benefits…But the officials must know where to offer the lands using scientific data, evidence‐based decisions. (FGD 5).

During transect walks and discussions, farmers revealed that the proposed MER is now occupied by solar power projects after further clearing of existing forest patches. They consider the solar power projects to be shortsighted approaches to land use planning as destruction of the remaining few elephant habitats this will exacerbate HEC:

Solar panels were established across 100s and 1000s of acres of the forest. (FGD 1)

While criticising the government's short‐sighted development planning, some discussants highlighted how past lessons do not inform present actions. They also highlighted that communication and consultation with local communities had been limited. Some villagers reported having no knowledge of the purpose of the developments or how they influence elephant movements, despite increasing experiences of negative interactions with elephants. The absence of community engagement and knowledge transfer suggests that past HEC experiences were not systematically used to inform present development planning.

Some people in this area have no idea of the development infrastructure in the area; they don't know and have never been to the Mattala airport or Hambantota harbour. Those infrastructures were developed to fulfil the needs of the rich rather than to satisfy the needs of the locals. (FGD 3)

### 3.2 Institutional and Structural Challenges

Walsapugala farmers shared an array of institutional barriers encountered when seeking support for HEC. These barriers were mostly related to compensation for the damages (e.g., property and livelihoods) caused by elephant conflicts. However, while highlighting the resource limitations related to some of those institutions, these farmers criticised the effectiveness of the existing strategies within those institutions to address HEC.

In an era where financial compensation is highlighted as a promising tool to mitigate human‐wildlife conflicts, Walsapugala farmers complain that compensation programs exist solely for paperwork. They did not know whom they should first report to after a conflict situation. The findings from across eight FGDs revealed different institutions as their first point of post‐HEC contact; for example, *Grama Sewa Niladari*, Mahaweli authority, wildlife officers, provincial council officers, were among these institutions. Wildlife officers were the most common first point of contact, especially during crop raiding and house damage by elephants, which is when the farmers usually first attempted to call them. Regardless of the severity of the situation, farmers complain that the officials fail to come to the village to offer the necessary assistance. Farmers consider filling out forms to obtain a significantly small compensation for the damages caused by elephant attacks a waste of time and money. To collect the required forms, complete them and return them, farmers must travel to Hambantota city which incurs fuel costs. Moreover, farmers complained that their applications for compensation are usually rejected because they lack documentation to prove their land ownership. Ultimately, the bureaucratic processes that require many documents hinder the accessibility of compensation programs, particularly for poor and remote farmers.

After we are attacked by an elephant, the Grama Sewa Niladari is coming to inspect the damage, and we will be entitled to get at least LKR10,000 more or less…‥, but we can't get that small amount even because we don't have ownership to this land, we are entitled as illegal residents, so we are asked to show proof of ownership to the land. It's impossible to get those ownership documents from the Mahaweli. We have been to the Mahaweli office uncountable times, and all of them were total frustrations. We have been here for over 10 years, but we don't have any ownership yet. When we were to receive compensation for the elephant, we were being blocked from that because of this ownership issue. (FGD 3)

After a crop‐raiding event, who comes first to check on us? It's the elephant we can tell first, to be honest. Sometimes, after we inform the wildlife officers, we are asked to go see the GA (government agent). We have not been paid compensation so far. But we are asked to fill out some forms. We have even visited the officers in person and requested compensation, but no luck. (FGD 4)

When farmers were discussing as more participants in FGDs shared their negative experiences regarding lack of access to, or delays in, compensation, for example, shared anger towards the officials was expressed. Some farmers used swear words about the situation, concerned about the corruption of some officials. However, several farmers pointed out that wildlife officers also face issues that affect their competency, such as those related to infrastructure. For example, the discussants revealed that wildlife officers lack motor vehicles to use during conflict events in the village, especially at night (which is when most crop raids occur). In one of the discussions (FGD 2), farmers mentioned that only 12 wildlife officers are employed across the whole Hambantota District. Moreover, the discussants further stated that the wildlife officers and other governing authorities do not understand the difficulties farmers face when trying to access elephant repellent strategies. They highlighted this in combination with the infrastructure‐related barriers that officers are also facing.

We have requested to issue elephant crackers to farmer associations, because since they issue it individual basis, when I go asking for it, they only issue two elephant crackers per day. To receive those two, it cost me a lot, and sometimes they even refuse to issue them, and we will have to come back empty‐handed. Sometimes it takes like five cracks per night. When we called the DWC (Department of Wildlife Conservation) and requested them to deliver us these essentials, like the elephant firecrackers, they said they don't have petrol to come. (FGD 5)

During a transect walk, farmers showed the water tanks where some elephants usually spend their daytime to ease the effects of the hot‐dry climate. These farmers are very observant of the whereabouts of particular elephants whom they identified as most frequently engaged in crop raiding activities. During the discussions, some farmers expressed disappointment with wildlife officers who are not supportive of reducing the threat of an elephant raid—which is usually done by chasing elephants identified as troublesome into forest patches before nightfall.

Very recently, there was an elephant in this tank (river), we all were watching it after informing the DWC to come and chase it away from the village. We were watching and confining it throughout the day while staying on the bank of the tank (river)…‥ Around 6.00 pm, what the officers did was just chase the elephant away to another nearby village and then walk away. Because after 6.00 pm, they again complain they can't see properly. (FGD 7)

Some discussants criticised the knowledge of the officials about elephant conflict issues and their short‐sighted strategies, such as chasing elephants away from conflict sites. According to these farmers, chasing elephants from one area to another is not an informed and inclusive process. Walsapugala farmers highlighted that the communities in both the area where elephants are chased from and where they are chased to must be aware of the activity and must have consented. Communities on the receiving end of this elephant chasing activity could end up with increased crop raiding, housing damage and human deaths due to the additional elephants in their area.

Farmers also acknowledged that, in some instances, although officials are not purposefully attempting to drive elephants into an adjacent village, high human density and limited remaining elephant habitat mean this is often the outcome. Therefore, this practice of chasing elephants between troubled areas just shifts the problem from one place to another rather than resolving it.

These farmers are concerned about the effectiveness of the existing solutions offered by the wildlife officers but also about their effects on elephants. The discussants were highly concerned about the repeated use of firecrackers on the well‐being of elephants. They were surprised that authorities pay no attention to the potential dangers of these traditional repellent strategies, which are still being promoted:

… When an elephant cracker is lit up, is it useful? It is dangerous for both humans and animals, as sound leads to hearing disabilities in many other animals. So, how come the DWC officers suggest that we use these destructive crackers? (FGD 8)

### 3.3 Power and Corruption

The barriers related to power and corruption were mostly related to the illegal activities taking place within the proposed MER and the lack of law enforcement against those activities. Not only are farmers aware of those illegal activities, but they are also concerned about the well‐being of the elephants inside the MER.

In all the FGDs, the farmers expressed frustration about illegal activities happening within the proposed MER and the bribery culture between illegal occupants and officials. Walsapugla farmers criticised bribery actions by the officials in their area, which they say have become a lifestyle for those officials.

Those officers receive vegetables and rice for their family functions for free through these illegal farmers, which is why they are never subjected to legal action by authorities. (FGD4)

The farmer communities are experiencing a power imbalance between those living outside the MER and the illegal occupants within the MER. They questioned why community members who live adjacent to the reserve and go into it to collect firewood are arrested but the illegal farmers monocropping inside the reserve are not.

But those large‐scale farmers, who are doing monocropping in those lands offered from MER, elephants don't even go near those fences, their farmlands are 50‐100 acres in extent. Their electric fences were so powerful that we had no resources or power to develop such fences. (FGD 2)

There are people with T56 guns within the MER now; go get a drone and pay a visit in that; you will see them. (FGD 6)

Farmers are also concerned about the treatment of elephants within the MER by the illegal merchants and about officials reactions. The illegal merchants who occupy the reserve use high‐voltage electric fences to protect their crop and infrastructure which can injure elephants. Farmers shared that even if an elephant died due to those illegal fences, the authorities do not take action against the illegal merchants.

… one elephant died in one of those lands, and we saw the DWC bring the corpse. In the evening, they came back and gave a bunch of elephant crackers to that merchant and told us that the autopsy of the elephant mentioned the cause of death as high blood pressure. But we saw that elephant with a wire around its neck. (FGD 8).

## 4 Discussion

Although the barriers to coexistence have been divided across themes (socio‐political drivers, institutional and structural challenges and power and corruption), our results revealed that they are interrelated. Therefore, attempts to understand and address the nuances of barriers to coexistence require interdisciplinary approaches supported by in‐depth qualitative research to capture context‐specific barriers that shape coexistence outcomes. Although interactions between farmers and wildlife mostly manifest as conflicts (Mekonen 2020), our study identified that those interactions are conflicts to coexist due to various latent challenges/issues, which we define as barriers to coexistence. Also, those struggles are mostly between farmers and governing/managing authorities of wildlife and protected areas and thus can be understood as human‐human (rather than human‐wildlife) conflicts. This is congruent with Mathotaarachchi et al. (2021) who describe human‐wildlife conflicts as a physical expression of socio‐political human‐human conflict that is influenced by existing social systems. Further, Dando et al. (2025) and Redpath et al. (2015), also argue that conservation challenges, such as stakeholder engagement in species reintroduction programs, are human‐human conflicts due to competing interests of different stakeholder groups. In our study, the interests of the farmers also reflected the needs and interests of the wildlife (especially elephants). Hence, this case study highlighted the need for a deeper understanding of coexistence before siloing the key stakeholders involved into morally defined conservation proponents or opponents. Our aim is not to distinguish or define such moral demarcations of stakeholders, but to give a nuanced understanding of the intricacies of existing barriers to coexistence.

Barriers to coexistence manifest in Walsapugla village as consequences of unsustainable development and conservation planning. This includes infrastructure development, such as highways, that impact the well‐being of wildlife. To overcome these, suggestions include conducting longitudinal biodiversity and roadkill surveys in areas with critical habitat and identifying locations for wildlife crossings, fences and traffic controls (Bennett 2017; Healey et al. 2020; Spencer et al. 2023). Simultaneously, the well‐being of the communities relocated for conservation or development objectives must also be prioritised (Rai 2019). Mekonen (2020), for example, suggests relocating agricultural activities out of national park boundaries to support peaceful coexistence between humans and wildlife. For the Walsapugala farmers, their well‐being was traded for development and then neglected after their relocation into conflict‐prone areas, where expanding human settlements intensified HWC. This is consistent with HWC in other parts of the world. For example, Turkiye anthropogenic activities surrounding protected areas, such as open‐garbage dumps, orchards and beehives, exacerbate human‐brown bear conflicts by increasing availability and ease of access to anthropogenic food sources (Sıkdokur et al. 2024). Since human presence is central for coexistence, effective habitat management becomes critical, with adaptive management approaches offering a key pathway for addressing the evolving nature of such conflicts.

Our study highlighted that community resettlement associated with development and conservation projects can generate compounded social and environmental vulnerabilities that influence the conditions for coexistence. This is consistent with many other parts of Sri Lanka as well as across Asia. Community resettlement programs for development infrastructure, such as coal power plants in Sri Lanka, have shown rising vulnerabilities to environmental pollution (Jayasinghe 2023). Pandey et al. (2025) show effects on health and social security experiences among community groups displaced for conservation purposes in Nepal's Terai Arc Landscape. Here, disruption of social relationships compounded by intermingling with diverse groups and the prolonged relocation process affects the mental well‐being of communities. The social security outcomes also varied between those relocated to urban areas, who experienced reduced HWC, and those relocated to remote areas, who continued to experience challenges with HWC.

The temporary conflict mitigation action of repelling wild elephants did not provide Walsapugala farmers with motivation to increase tolerance of elephant encounters. Walsapugala farmers had shown special consideration for the existing strategies to repel elephants, especially strategies encouraged by the wildlife officers, such as chasing conflicting elephants to nearby forest patches. Farmers identified this as shifting the problem from one location to another rather than resolving it. This is consistent with the findings of Matsuura et al. (2024), who observed that elephants returned to raid crops shortly after being chased in Central African forest landscapes. However, Cabral de Mel et al. (2022), suggest that chasing problem elephants away using automated early warning systems results in effective aversive conditioning, where elephants learn to associate a particular behaviour with unpleasant stimuli.

Although compensation for damage caused by wildlife is essential for promoting HW‐coexistence (Ma et al. 2024), HWC persists—suggesting that compensation strategies need reconsidering. In Nepal's Gaurishankar Conservation Area, for example, conflicts involving leopards and Himalayan black bears continue to rise despite improvements in compensation policies, which potentially reflect increased reporting of attacks (Pathak et al. 2024). To address this, Pathak et al. (2024) recommend strengthening alternative livelihood options to promote coexistence. Considering this, we argue that improved compensation policies cannot be solely attributed to mitigating HWC since the number of conflicts continues to increase and then call for alternative livelihoods instead. However, improved compensation policies may result in a comprehensive assessment of HWC. While focusing on addressing rising conflicts, our study highlighted the significance of understanding the underlying barriers to conflict mitigation strategies such as compensations.

Findings from Walsapugala village revealed barriers to effective compensation processes due to numerous reasons including bureaucratic procedures, inadequate payouts and inefficiencies in damage evaluations which again have parallels in other parts of the world. Focusing on Koshi Tappu Wildlife Reserve (KTWR) in Nepal, Dahal et al. (2025, 1) highlighted that local communities perceive ‘corruption, procedural hassles, misuse of political authority and biased treatment’ as key barriers to effective compensation and that a proper damage evaluation system and relief policy is needed to promote HW‐coexistence. Moreover, Wang et al. (2024) identified the non‐congruency between the approval of compensation and effective prevention measures. Consistent with the findings from Walsapugala, lack of farmer awareness about the pathways and tools to apply for compensation has been identified in China, Nepal and Kenya (Rodriguez 2008; Neupane et al. 2018; Wang et al. 2024). Bulte and Rondeau (2005), considering a wide range of species, including elephants, rhinos and wolves, identified mixed evidence of success for compensation programs across the globe. Evidence from Northern America at least partially attributes the success of reintroduction and conservation of wolves and brown bears to effective compensation programs. Compensation efforts in regions with less secure property rights and ineffective administrative controls are more likely to fail, especially in the African region (Bulte and Rondeau 2005).

For Walsapugala farmers, the next issue, following the absence of compensation for conflicting encounters, was the lack of market opportunities to sell their crops. Community‐based conservation studies underscore the importance of developing synergies between conservation and agriculture as incentives for coexistence (Naidoo et al. 2015; Drake et al. 2020; Meyer and Borner 2022). For instance, when conservation and agriculture are economically interdependent, the opportunity costs of agriculture may equal the returns from conservation. Naidoo et al. (2015) suggest such economic interdependencies where South African households benefit through consumptive and non‐consumptive tourism. However, the potential of such economic models to adequately compensate for actual losses incurred from wildlife interactions, particularly in contexts with restricted access to the tourism market, is questionable. This is because the tourism benefits are often unevenly distributed and may not directly correspond to household‐level losses (Zhang et al. 2025).

According to Zimmermann et al. (2020), there are three levels of conflict over wildlife starting with level 1 disputes over losses of crops, livestock, income and safety. Level 2, underlying conflict, occurs when the level 1 issues are not resolved satisfactorily and reoccur. This progresses to level 3, deep‐rooted conflict when the recurring issues not satisfactorily resolved and social identity or values are threatened. Of these three levels, the conflict situation in Walsapugala fluctuates between levels 2 and 3. All the respondents in this study complained about ongoing illegal activities inside the MER and the lack of legal consequences for offenders. Further, amidst the prolonged compensation failures and repeated damage to crops and houses due to elephant attacks resulted in broader grievances and tensions over their farming identities, Walsapugala farmers question why they are treated differently. This revealed a conflict between farmers and authorities rather than farmers and elephants. However, Rigg et al. (2011) showed that these deep‐rooted conflicts could result in perpetuated hatred towards the problematic wildlife (i.e., wolves in Slovakia even after sheep depredation had reduced to a negligible level). Hence, for level 3 conflict conditions, Treves et al. (2013) emphasise that financial compensation would not improve the tolerance towards the conflict wildlife since the deeply held values between stakeholders have drastically deteriorated. To achieve conservation objectives, an exclusive focus on implementing technical solutions to conflict mitigation will not be effective unless approaches are in place to recognise the long‐overlooked hidden barriers to coexistence. Such approaches can help overcome socio‐political and institutional barriers by strengthening social trust and governance systems alongside technical and financial interventions.

If coexistence is the tolerance of communities towards wildlife, how would communities strive to tolerate amidst these socio‐political and institutional barriers, and what would they receive in return for doing so? Compensation for wildlife encroachment damage is mostly implemented as a HWC mitigation strategy. However, promoting isolated technical and financial solutions do not necessarily support coexistence as a meaningful conservation outcome as such approaches tend to overlook the hidden barriers to coexistence. Because communities have deep‐rooted values related to interacting with wildlife (in this case, elephants), they are unwilling to compromise their wellbeing or others. For instance, Walsapugala farmers, while complaining about crop raiding by elephants and lack of support from wildlife officers, show consideration for the well‐being of the elephants, their lost habitat and lack of food sources. Therefore, the farmer communities are not waiting for anthropocentric solutions and are unwilling to settle for short‐term solutions such as financial compensation and electric fencing to repel elephants. Our study revealed an array of underlying issues that act as barriers for farmer communities to continue their livelihoods in the presence of elephants. Hence, if conservation only targets implementation of conflict mitigation strategies (e.g., financial compensation and electric fencing), the intricate and interrelated webs of issues underlying them will remain unseen, leaving coexistence an impracticable target in conservation.

Farmers in Walsapugala, despite experiencing crop losses and insufficient institutional support, demonstrated concern for the well‐being of elephants and acknowledged constraints such as habitat loss and declining food availability. These perspectives reveal that farmer communities are not passive recipients of anthropocentric solutions and do not view temporary measures such as financial compensation or electric fencing as sufficient. Instead, our study highlights multiple underlying challenges that constrain livelihoods alongside elephants, underscoring the importance of actively involving local communities in the design and implementation of conservation strategies to ensure that all barriers to coexistence are identified and addressed. Moreover, these findings emphasise the need for adaptive management approaches that can respond to hidden barriers that may arise during implementation, such as corruption, inequitable resource distribution, or institutional capacity limitations. Without such community engaged and adaptive processes, conservation efforts that focus narrowly on conflict mitigation risk overlooking the complex and interrelated barriers that continue to render coexistence an impracticable conservation goal.

## 5 Conclusion

The ethnographic study revealed barriers to coexistence with elephants across three themes: socio‐political drivers, institutional and structural challenges and power and corruption. The study demonstrated the interrelatedness and complexity of these barriers, portraying a nuanced understanding of them. We discovered various other forms of conflicts that extend beyond farmer‐elephant interactions and influence barriers to coexistence. For instance, conflicts between farmers and the wildlife governing authorities and between farmers and other wildlife. Moreover, perpetuated issues of farmers, such as the absence of proper land rights, remained unresolved for decades.

Coexisting with elephants is not novel to the Walsapugala farmers; however, the conditions in which they coexist have changed due to increasingly complex and interrelated barriers. We argue that future research into coexistence must move beyond an exclusive focus on implementing technical solutions for conflict mitigation. A meaningful pathway towards coexistence requires a deeper understanding of the hidden barriers to coexistence, such as those that are revealed from our study (e.g., corruption and institutional and structural challenges) that prevent effective implementation of conflict mitigation strategies.

To achieve this, given the long‐standing relationships that farmer communities hold with wildlife, conservation practitioners and HWI governing authorities must actively involve local communities in the design and implementation of conservation strategies to ensure that barriers to coexistence are recognised and addressed. For such an approach, adaptive management approaches are essential to recognise and respond to the barriers arising during the implementation of those strategies towards conservation to remain effective and just.

## Author Contributions

**Anuradhi Dulangi Jayasinghe:** conceptualization (lead), data curation (lead), formal analysis (lead), methodology (lead), project administration (lead), writing – original draft (lead). **Georgette Leah Burns:** conceptualization (equal), funding acquisition (equal), methodology (supporting), project administration (lead), supervision (lead), writing – review and editing (lead). **Duan Biggs:** conceptualization (supporting), funding acquisition (equal), project administration (supporting), supervision (supporting), writing – review and editing (supporting).

## Funding

This work was supported by, Griffith University, the Rufford Foundation (43247‐1), and the Olajos Goslow Chair at Northern Arizona University.

## Conflicts of Interest

The authors declare no conflicts of interest.

## Data Availability

All data used for the qualitative analysis are presented in the results sections and in the Appendices A, B, C (anonymous and coded).

## Appendix A Discussion Framing Summary

Since we used an inductive approach, our questions were not tied to any preconceived theories/notions. The questions raised during focus group discussions, transect walks and participant observations are open‐ended, but they all had a common focus on the research questions of the study. The focus group discussions followed the following pattern of questions:

1. How long have you all been here in this area?
2. What differences do you all notice then and now?
3. How about elephant conflicts, then and now?
4. Who would you first reach after an elephant attack/conflict?
5. What are the available support systems in such situations?
6. What are your strategies to avoid conflicts with elephants?
7. Will your kids continue farming?
8. What do you think we could do about this issue?

## Appendix B Coding Illustration

Files = Number of focus group discussions, References = Number of encounters throughout all the FGDs.

## Appendix C Coding Illustration for Specific Themes

**Table:**

Codes	Remarks	Themes
Unsustainable development planning by the government	Development is not for locals in the area but for anyone else outside. Large‐scale infrastructure projects like the Hambantota Port, Mattala Airport and highways.	Socio political drivers
Non‐inclusive decision making	The locals were not invited during the decision‐making stage, but they have been gathered by the then local politicians when taking away the local lands for the development projects. According to the villagers, during those gatherings (discussions) the villagers were convinced about getting new job opportunities from the development thus they had given away their lands to the government.	
Land rights issues	No declared deeds (no legitimate lands), This also includes the lack of proper demarcation of government owned structured in the village such as the grazing grounds for cattles. Villagers openly accepting that they grabbed the lands.	
Issues related to livelihoods	This includes other issues, such as the lack of market opportunities for the villagers' productions. Also, farmers become labourers on other lands and abandon their lands, ultimately ending as patches for wild elephants. This again increases the conflicts with elephants.	
Issues from other wildlife	This includes the conflicts between the toque macaque (Rilawa) and farmers and other wildlife (Peacock, porcupines, etc.). This also includes problems from other captive‐bred animals like cattle.	
Institutional relationships	People are unaware/confuse of the institutional hierarchy to reach in an event of a conflict. Even reaching the institution in search of support to survive amidst of the conflict isn't helpful as the villages must keep visiting those institutions.	
Ineffective and malfunctioning compensation programs	By the government, or other parties outside the village.	Institutional and structural challenges
Infrastructure related barriers	Resource barriers in general from the government side.	
Knowledge of the officials about elephants and strategies to repel them	As a constraint the fact that they don't have enough knowledge or practical knowledge. Not only about elephants but also about strategies like the use of electric fences. Knowledge about strategies to repel elephants.	
Power dynamics	Legal pluralism also included.	Power and corruption
Actions (illegal) of powerful farmers	The torturing from the farmers who are illegally cultivating inside the proposed Managed Elephant Reserve. The power of these illegal farmers supersedes that of these community members.	
Corruption and bribery	Victims of the elephant conflicts must bribe the officials responsible for the compensations to get some level of an action for the damages caused by the elephants.	

## References

[ece373859-bib-0002] Badola, R. , T. Ahmed , A. K. Gill , et al. 2021. “An Incentive‐Based Mitigation Strategy to Encourage Coexistence of Large Mammals and Humans Along the Foothills of Indian Western Himalayas.” Scientific Reports 11, no. 1: 5235. 33664314 10.1038/s41598-021-84119-7 PMC7933403

[ece373859-bib-0003] Begum, T. 2024. Death Tolls Mount as Elephants and People Compete for Land in Sri Lanka. Guardian. https://www.theguardian.com/environment/2024/mar/19/sri‐lanka‐elephants‐people‐deaths‐natural‐resources‐climate‐crisis‐coexistence‐aoe.

[ece373859-bib-0005] Bennett, V. J. 2017. “Effects of Road Density and Pattern on the Conservation of Species and Biodiversity.” Current Landscape Ecology Reports 2: 1–11. 10.1007/s40823-017-0020-6.

[ece373859-bib-0006] Bhandari, A. , S. Bagale , T. Silwal , and M. Paudel . 2020. “Spatio‐Temporal Patterns of Wildlife Attacks on Humans in Chitwan National Park, Nepal.” Scientific Reports in Life Sciences 1, no. 1: 1–20.

[ece373859-bib-0007] Braun, V. , and V. Clarke . 2019. “Reflecting on Reflexive Thematic Analysis.” Qualitative Research in Sport, Exercise and Health 11, no. 4: 589–597. 10.1080/2159676X.2019.1628806.

[ece373859-bib-0008] Bulte, E. H. , and D. Rondeau . 2005. “Why Compensating Wildlife Damages May be Bad for Conservation.” Journal of Wildlife Management 69, no. 1: 14–19. https://www.jstor.org/stable/3803581.

[ece373859-bib-0009] Bulte, E. H. , and D. Rondeau . 2007. “Compensation for Wildlife Damages: Habitat Conversion, Species Preservation and Local Welfare.” Journal of Environmental Economics and Management 54, no. 3: 311–322. 10.1016/j.jeem.2007.02.003.

[ece373859-bib-0010] Byrne, D. 2021. “A Worked Example of Braun and Clarke's Approach to Reflexive Thematic Analysis.” Quality & Quantity 56: 1391–1412. 10.1007/s11135-021-01182-y.

[ece373859-bib-0011] Cabral de Mel, S. J. , S. Seneweera , R. K. de Mel , et al. 2022. “Current and Future Approaches to Mitigate Conflict Between Humans and Asian Elephants: The Potential Use of Aversive Geofencing Devices.” Animals (Basel) 12, no. 21: 2965. 10.3390/ani12212965. 36359089 PMC9653792

[ece373859-bib-0013] Centre for Environmental Justice (CEJ) . 2024. “Proposal to Mitigate the Human‐Elephant Conflict in Sri Lanka.” https://slltsunamicharity.org/wp‐content/uploads/2024/08/Proposals‐to‐mitigate‐Human‐Elephant‐Conflict‐in‐Sri‐Lanka‐E‐book.pdf.

[ece373859-bib-0015] Dahal, N. K. , K. Harada , S. Adhikari , and R. Gurung . 2025. “Enhancing Human–Wildlife Coexistence: Community Perceptions of Compensation Policies in a Protected Area of Nepal.” GeoJournal 90: 91. 10.1007/s10708-025-11341-5.

[ece373859-bib-0016] Dando, T. R. , S. L. Crowley , R. P. Young , S. P. Carter , H. Denman , and R. A. McDonald . 2025. “Understanding Farmers' Perspectives and Engagement With Wildlife Conservation Practices: Insights From a European Wildcat Reintroduction.” People and Nature 7: 1987–2001. 10.1002/pan3.70070.

[ece373859-bib-0017] Drake, M. D. , J. Salerno , R. E. Langendorf , et al. 2020. “Costs of Elephant Crop Depredation Exceed the Benefits of Trophy Hunting in a Community‐Based Conservation Area of Namibia.” Conservation Science and Practice 68: e345. 10.1111/csp2.345.

[ece373859-bib-0019] Frank, B. F. , and J. A. Glikman . 2019. “Human‐Wildlife Conflicts and the Need to Include Coexistence.” In Human–Wildlife Interactions: Turning Conflict Into Coexistence, edited by B. F. Frank , J. A. Glikman , and S. Marchini . Cambridge University Press. https://books.google.com.au/books?hl=en&lr=&id=G26MDwAAQBAJ&oi=fnd&pg=PA1&dq=the+rhetoric+of+human+wildlife+coexistence&ots=m5u_USrwBE&sig=2kLl8f0eDYyoq7QZoufK_1jvC0M#v=onepage&q=the%20rhetoric%20of%20human%20wildlife%20coexistence&f=false.

[ece373859-bib-0020] Gao, Y. , A. Lambert , and S. Clark . 2023. “Grand Strategy for Human–Wildlife Coexistence.” Frontiers in Ecology and the Environment 21, no. 7: 308–309. 10.1002/fee.2668.

[ece373859-bib-0022] Ghosh, N. , L. Theengh , and P. Shrestha . 2023. “A Study on the Profile of a Forest Fringe Village in Pangolakha Wildlife Sanctuary, Sikkim, Facing Human‐Wildlife Conflict.” Sustainability and Biodiversity Conservation 3, no. 2: 26–40.

[ece373859-bib-0023] Gunawansa, T. D. , K. Perera , A. Apan , and N. K. Hettiarachchi . 2023. “The Human‐Elephant Conflict in Sri Lanka: History and Present Status.” Biodiversity and Conservation 32: 3025–3052. 10.1007/s10531-023-02650-7.

[ece373859-bib-0025] Healey, R. M. , J. R. Atutubo , M. D. Kusrini , et al. 2020. “Road Mortality Threatens Endemic Species in a National Park in Sulawesi, Indonesia.” Global Ecology and Conservation 24: e01281. 10.1016/j.gecco.2020.e01281.

[ece373859-bib-0026] Hemminger, K. , L. Eriksson , L. Nilsson , et al. 2025. “Farmers' Tolerance for Crop Damage Caused by Wildlife: The Role of Compensation.” Wildlife Biology 2025, no. 4: e01243. 10.1002/wlb3.01243.

[ece373859-bib-0028] IUCN . 2008. “IUCN (International Union for Conservation of Nature) 2008. *Elephas maximus* .” The IUCN Red List of Threatened Species. Version 2019‐3. https://www.iucnredlist.org/species/7140/12828813#geographic‐range.

[ece373859-bib-0029] Jayasinghe, A. D. 2023. “A Local Perspective of the Socio‐Environmental Vulnerability to Environmental Pollution and Economic Crises: A Case of Locals Around a Coal Power Plant in Sri Lanka.” Environment, Development and Sustainability 26: 5431–5450. 10.1007/s10668-022-02893-4. PMC9838292 36687735

[ece373859-bib-0031] Jolly, H. , and A. Stronza . 2024. “Insights on Human−Wildlife Coexistence From Social Science and Indigenous and Traditional Knowledge.” Conservation Biology 39: e14460. 10.1111/cobi.14460. PMC11959325 40165698

[ece373859-bib-0033] Kansky, R. , M. Kidd , and J. Fischer . 2020. “Does Money “Buy” Tolerance Toward Damage‐Causing Wildlife?” Conservation Science and Practice 3, no. 3: 1–16. 10.1111/csp2.262.

[ece373859-bib-0035] Ma, Z. , J. Li , R. Chen , X. Wei , and W. Chen . 2024. “Estimating the Impact of Wildlife Damage Compensation Policy on Farmers' Incomes.” Journal for Nature Conservation 81: 126709. 10.1016/j.jnc.2024.126709.

[ece373859-bib-0036] Madden, F. , and B. McQuinn . 2015. “Understanding Social Conflict and Complexity in Marine Conservation.” In Human‐Wildlife Conflict, edited by M. M. Draheim , F. Madden , J.‐B. McCarthy , and C. Parsons , 3–16. Oxford University Press.

[ece373859-bib-0037] Mahaweli Authority of Sri Lanka (MASL) . 2021. “Annual Report – 2021, Parliament of Sri Lanka.” https://www.parliament.lk/uploads/documents/paperspresented/1691571939025956.pdf.

[ece373859-bib-0038] Mariyathas, S. , N. Perera , and M. Yehiya . 2016. “What Development Has Done to a Town: Lessons From Hambantota, Sri Lanka, Bhumi.” Planning Research Journal 5, no. 1: 57–72. 10.4038/bhumi.v5i1.24.

[ece373859-bib-0039] Mathotaarachchi, K. P. , W. M. C. P. Godage , and K. A. A. N. Thilakarathna . 2021. “From Conflict to Coexistence: A Critical Look at Issues Related to Human‐Wildlife Interactions in Sri Lanka.” Asian Journal of Law and Governance 3, no. 1: 47–59.

[ece373859-bib-0040] Matsuura, N. , M. Nomoto , S. Terada , C. M. Yobo , H. R. Memiaghe , and G.‐M. Moussavou . 2024. “Human‐Elephant Conflict in the African Rainforest Landscape: Crop‐Raiding Situations and Damage Mitigation Strategies in Rural Gabon.” Frontiers in Conservation Science 5: 1356174. 10.3389/fcosc.2024.1356174.

[ece373859-bib-0041] Mekonen, S. 2020. “Coexistence Between Human and Wildlife: The Nature, Causes and Mitigations of Human Wildlife Conflict Around Bale Mountains National Park, Southeast Ethiopia.” BMC Ecology 20: 51. 10.1186/s12898-020-00319-1. 32928171 PMC7489024

[ece373859-bib-0042] Meyer, M. , and J. Borner . 2022. “Rural Livelihoods, Community‐Based Conservation, and Human–Wildlife Conflict: Scope for Synergies?” Biological Conservation 272: 109666. 10.1016/j.biocon.2022.109666.

[ece373859-bib-0044] Naidoo, R. , L. C. Weaver , R. W. Diggle , G. Matongo , G. Stuart‐Hill , and C. Thouless . 2015. “Complementary Benefits of Tourism and Hunting to Communal Conservancies in Namibia.” Conservation Biology 30, no. 3: 628–638. 10.1111/cobi.12643. 26537845

[ece373859-bib-0045] Neupane, B. , S. Budhathoki , and B. Khatiwoda . 2018. “Human‐Elephant Conflict and Mitigation Measures in Jhapa District, Nepal.” Journal of Forest and Livelihood 16, no. 1: 103–112. 10.3126/jfl.v16i1.22885.

[ece373859-bib-0047] Oberlack, C. , D. Sietz , E. Bürgi Bonanomi , et al. 2019. “Archetype Analysis in Sustainability Research: Meanings, Motivations, and Evidence‐Based Policy Making.” Ecology and Society 24, no. 2: art26. 10.5751/ES-10747-240226.

[ece373859-bib-0048] Pacheco‐Romero, M. , T. Kuemmerle , C. Levers , D. Alcaraz‐Segura , and J. Cabello . 2021. “Integrating Inductive and Deductive Analysis to Identify and Characterize Archetypical Social‐Ecological Systems and Their Changes.” Landscape and Urban Planning 215: 104199. 10.1016/j.landurbplan.2021.104199.

[ece373859-bib-0049] Pandey, H. P. , T. N. Maraseni , and A. Apan . 2025. “Resettlement for Conservation: Assessing Health and Social Security Challenges in Nepal's Biodiverse Regions.” Global Transitions 7: 247–261. 10.1016/j.glt.2025.04.006.

[ece373859-bib-0050] Paranage, K. 2019. “The Mahaweli Development Project and the ‘Rendering Technical’ of Agrarian Development in Sri Lanka.” Heliyon 5, no. 6: e01811. 10.1016/j.heliyon.2019.e01811. 31194081 PMC6551383

[ece373859-bib-0051] Pathak, A. , S. Lamichhane , M. Dhakal , et al. 2024. “Human‐Wildlife Conflict at High Altitude: A Case From Gaurishankar Conservation Area, Nepal.” Ecology and Evolution 14, no. 7: e11685. 10.1002/ece3.11685. 39224839 PMC11367734

[ece373859-bib-0052] Perera, Y. 2021. “Human‐Elephant Conflict Reaching Climax: A Jumbo Problem Without Solution.” The Morning. https://www.themorning.lk/articles/124327.

[ece373859-bib-0054] Pooley, S. , S. Bhatia , and A. Vasava . 2020. “Rethinking the Study of Human–Wildlife Coexistence.” Conservation Biology 35, no. 3: 784–793. 10.1111/cobi.13653. 33044026 PMC8246872

[ece373859-bib-0055] Rai, J. 2019. “Displacement Versus co‐Existence in Human Wildlife Conflict Zones: An Overview.” Journal of Geography, Environment and Earth Science International 19, no. 4: 1–16. 10.9734/jgeesi/2019/v19i430093.

[ece373859-bib-0056] Rathnayake, C. W. M. , S. Jones , M. Soto‐Berelov , and L. Wallace . 2022. “Assessing Protected Area Networks in the Conservation of Elephants ( *Elephas maximus* ) in Sri Lanka.” Environmental Challenges 9: 100625. 10.1016/j.envc.2022.100625.

[ece373859-bib-0057] Redpath, S. M. , S. Bhatia , and J. Young . 2015. “Tilting at Wildlife: Reconsidering Human–Wildlife Conflict.” Oryx 49: 222–225. 10.1017/S0030605314000799.

[ece373859-bib-0058] Register General's Department Sri Lanka . 2023. “Mid‐Year Population Estimates by District & Sex, 2014–2023 (Contd.).” http://www.statistics.gov.lk/Resource/en/Population/Vital_Statistics/Mid‐year_population_by_district.pdf.

[ece373859-bib-0059] Rigg, R. , S. Findo , M. Wechselberger , M. L. Gorman , C. Sillero‐Zubiri , and D. W. Macdonald . 2011. “Mitigating Carnivore‐Livestock Conflict in Europe: Lessons From Slovakia.” Oryx 45: 272–280.

[ece373859-bib-0060] Rodrigo, M. 2021. “Sri Lanka Seeks Peace With Pachyderms as Human‐Elephant Conflicts Escalate, Mongabay.” https://news.mongabay.com/2021/07/sri‐lanka‐seeks‐peace‐with‐pachyderms‐as‐human‐elephant‐conflicts‐escalate/.

[ece373859-bib-0061] Rodriguez, S. L. 2008. “Perceptions and Attitudes of a Maasai Community Regarding Wildlife‐Damage Compensation, Conservation, and the Predators That Prey on Their Livestock.” Human Dimensions of Wildlife 13, no. 3: 205–206. 10.1080/10871200801886137.

[ece373859-bib-0064] Schwartz‐Shea, P. , and D. Yanow . 2012. Interpretive Research Design: Concepts and Processes. Taylor & Francis Group, ProQuest Ebook Central. https://ebookcentral‐proquest‐com.libraryproxy.griffith.edu.au/lib/griffith/detail.action?docID=957663.

[ece373859-bib-0065] Shazuli, H. 2023. “One Elephant a Day: Sri Lanka Wildlife Conflict Deepens as Death Toll Rises, Mongabay.” https://news.mongabay.com/2023/05/one‐elephant‐a‐day‐sri‐lanka‐wildlife‐conflict‐deepens‐as‐death‐toll‐rises/.

[ece373859-bib-0066] Sıkdokur, E. , M. Naderi , E. Çeltik , et al. 2024. “Human‐Brown Bear Conflicts in Türkiye Are Driven by Increased Human Presence Around Protected Areas.” Ecological Informatics 81: 102643.

[ece373859-bib-0067] Spencer, K. L. , N. J. Deere , M. Aini , et al. 2023. “Implications of Large‐Scale Infrastructure Development for Biodiversity in Indonesian Borneo.” Science of the Total Environment 866: 161075. 10.1016/j.scitotenv.2022.161075. 36565871

[ece373859-bib-0070] Treves, A. , L. Naughton‐Treves , and V. Shelley . 2013. “Longitudinal Analysis of Attitudes Toward Wolves.” Conservation Biology 27: 315–323. 23293913 10.1111/cobi.12009

[ece373859-bib-0071] Treves, A. , and F. J. Santiago‐Avila . 2020. “Myths and Assumptions About Human‐Wildlife Conflict and Coexistence.” Conservation Biology 34: 811–818. 10.1111/cobi.13472. 32406969

[ece373859-bib-0072] Wahab, M. K. A. , O. Komolafe , and A. Adewumi . 2021. “Assessment of Human‐Wildlife Conflicts in Idanre Forest Reserve, Ondo State.” Nigeria. Scientific Reports in Life Sciences 2, no. 2: 20–29.

[ece373859-bib-0073] Wang, W. , T. Wronski , and L. Yang . 2024. “The Status of Wildlife Damage Compensation in China.” Animals 14, no. 2: 292. 10.3390/ani14020292. 38254461 PMC10812642

[ece373859-bib-0074] Wickramasinghe, K. 2024. “HEC in Hambantota Walsapugala Farmers Urge Authorities to Release MER for Elephants.” Daily Mirror Online. https://www.dailymirror.lk/plus/HEC‐in‐Hambantota‐Walsapugala‐farmers‐urge‐authorities‐to‐release‐MER‐for‐elephants/352‐294246.

[ece373859-bib-0076] Yeshey, Y. , R. J. Keenan , R. M. Ford , and C. R. Nitschke . 2024. “Social and Ecological Dimensions Are Needed to Understand Human‐Wildlife Conflict in Subsistence Farming Context.” People and Nature 6, no. 6: 2602–2617. 10.1002/pan3.10740.

[ece373859-bib-0077] Zhang, Y. , P. Hanna , and F. Vanclay . 2025. “Social Impacts From Tourism Development in Protected Areas: Comparing Communities in Different Conservation Zones of Wulingyuan World Heritage Site, China.” Land Use Policy 158: 107753. 10.1016/j.landusepol.2025.107753.

[ece373859-bib-0078] Zimmermann, A. , B. McQuinn , and D. W. Macdonald . 2020. “Levels of Conflict Over Wildlife: Understanding and Addressing the Right Problem.” Conservation Science and Practice 2: e259. 10.1111/csp2.259.

